# Effects of Enteral Nutrition on Patients With Oesophageal Carcinoma Treated With Concurrent Chemoradiotherapy: A Prospective, Multicentre, Randomised, Controlled Study

**DOI:** 10.3389/fonc.2022.839516

**Published:** 2022-02-25

**Authors:** Jiahua Lyu, Anhui Shi, Tao Li, Jie Li, Ren Zhao, Shuchai Zhu, Jianhua Wang, Ligang Xing, Daoke Yang, Conghua Xie, Liangfang Shen, Hailin Zhang, Guangying Zhu, Jing Wang, Wenyan Pan, Fang Li, Jinyi Lang, Hanping Shi

**Affiliations:** ^1^Sichuan Cancer Hospital, School of Medicine, University of Electronic Science and Technology of China, Chengdu, China; ^2^Department of Radiotherapy, Peking University Cancer Hospital, Beijing, China; ^3^Department of Radiotherapy, Shanxi Provincial Cancer Hospital, Taiyuan, China; ^4^Department of Radiotherapy, General Hospital of Ningxia Medical University, Yinchuan, China; ^5^Department of Radiotherapy, Fourth Hospital of Hebei Medical University, Shijiazhuang, China; ^6^Department of Radiotherapy, Henan Provincial Cancer Hospital, Zhengzhou, China; ^7^Department of Radiotherapy, Shandong Cancer Hospital, Shandong University, Jinan, China; ^8^Department of Radiotherapy, First Affiliated Hospital of Zhengzhou University, Zhengzhou, China; ^9^Department of Oncology, Zhongnan Hospital, Wuhan University, Wuhan, China; ^10^Department of Oncology, Xiangya Hospital, Central South University, Changsha, China; ^11^Department of Gastrointestinal Surgery/Clinical Nutrition, Beijing Shijitan Hospital, Capital Medical University, Beijing, China

**Keywords:** oesophageal carcinoma, enteral nutrition, chemoradiotherapy, nutritional status, prognosis

## Abstract

**Background:**

The oesophageal carcinoma patients show high incidence of malnutrition, which negatively affects their therapy outcome. Moreover, benefits of enteral nutrition remain to be studied in details in these patients. Therefore, we set to assess the effects of enteral nutrition on the nutritional status, treatment toxicities and survival in the oesophageal carcinoma patients treated with concurrent chemoradiotherapy (CCRT).

**Materials and Methods:**

Eligible patients were randomly assigned to either the experimental or control group. The patients in the experimental group were treated with a whole-course enteral nutrition management, while the control group were provided a unsystematic nutrition without setting intake goals for energy and protein. The primary endpoint was a change in body weight, while the secondary endpoints included nutrition-related haematological indicators, toxicities, completion rate of treatment and survival.

**Results:**

A total of 222 patients were randomised to either the experimental (n=148) or control (n=74) group. Patients in the experimental group showed significantly less decrease in body weight, serum albumin and haemoglobin levels, a lower incidence rates of grade ≥3 myelosuppression and infection, and a higher completion rate of CCRT than those in the control group. While analyses of the 2 and 3 year overall survival (OS) and progression-free survival (PFS) did not reveal differences between these groups, we observed a significantly higher OS at 1 year (83.6% vs. 70.0%). In the subgroup analysis, patients with patient-generated subjective global assessment (PG-SGA)=C were likely to have better OS and PFS with enteral nutrition.

**Conclusions:**

In EC patients treated with CCRT, enteral nutrition conferred positive effects on the nutritional status, treatment toxicities and prognosis, which mandate its inclusion in clinical practice.

**Clinical Trial Registration:**

This prospective trial has been registered with www.clinicaltrials.gov as NCT02399306.

## Introduction

Oesophageal cancer (EC) is a malignant tumour with a high incidence rate and more than 570000 cases are newly diagnosed worldwide every year (1). In China, the incidence rate of oesophageal cancer ranks fifth in men and ninth in women (2). Chemoradiotherapy is an important intervention for patients with oesophageal cancer (3).

The incidence of weight loss and malnutrition is high in patients with oesophageal cancer due to dysphagia, painful swallowing, alterations in metabolism, and adverse effects of radiotherapy and chemotherapy. Moreover, oesophageal carcinoma often ranks first in the incidence of malnutrition as 60–85% patients show different degrees of malnutrition (4–6). Furthermore, malnutrition not only reduces sensitivity to chemoradiotherapy, clinical outcomes and quality of life, but also increases treatment toxicity and hospital stays (7–9).

The European Society for Parenteral and Enteral Nutrition have suggested the importance of nutritional interventions in cancer patients (10). Moreover, recent studies have indicated that nutritional treatment can improve the nutritional status, treatment tolerability and quality of life, and decrease the treatment toxicity and duration of hospital stay in patients with esophageal cancer (11, 12).

However, well-designed, large-scale, and multicentre randomised studies need to be conducted. Additionally, previous studies have entirely focused on studying the effects of enteral nutrition in improving the body weight or other nutritional indicators, and only few studies have addressed the long-term analysis of patient survival. Therefore, the controversies pertaining the survival benefits of nutritional therapy remain to be studied.

The present study has its genesis in the discussion about a possibility of the beneficial effect of enteral nutrition on patients with esophageal cancer (EC). This is the first, prospective, multicentre, randomised, controlled clinical study in China and abroad, where the effect of enteral nutrition was evaluated in patients with EC undergoing chemoradiotherapy. The aim of the study was to evaluate the effects of enteral nutrition on nutritional status and treatment toxicities. Additionally, we performed a long-term follow-up of patients post-discharge to evaluate whether the administration of enteral nutrition can influence the survival.

## Materials and Methods

### Eligibility Criteria

Patients were recruited based on the following inclusion criteria: (1) histologically confirmed, stage II–III oesophageal carcinoma; (2) adequate digestive and absorption functions; (3) 18 years ≤ age ≤ 75 years; (4) the patient-generated subjective global assessment (PG-SGA) scores ≥ 2 points; (5) Karnofsky performance status (KPS) scores ≥ 70 points; (6) adequate haematological, renal, hepatic and pulmonary functions (defined as, absolute neutrophil count ≥ 1500 cells/mm^3^, a platelet count ≥ 100000 cells/mm^3^, haemoglobin levels ≥ 9.0 g/dL, bilirubin levels ≤ 1.5 times the upper limit of the institutional normal range, transaminase levels ≤ 3 times the upper normal limit and serum creatinine levels ≤ 2.0 mg/dL); and (7) no signs of perforation.

Further, the exclusion criteria were: (1) the intestinal functions severely impaired or patients intolerant to enteral nutrition; (2) incapable of oral feeding and insertion of nutrition tube or unwilling to accept the insertion of nutrition tube; (3) no malnutrition or nutritional risk; (4) severe malnutrition (weight loss > 10% or serum albumin [ALB] < 30 g/L or BMI < 18.5 kg/m^2^ or haemoglobin < 90 g/L) before the treatment; (5) serious heart, lung, liver and kidney diseases; (6) mental disease or severe cognitive disorder.

All participants provided written informed consent before participating in the study. The study protocol was approved by the Ethics Committee of our hospitals. Research was conducted in accordance with the 1964 Declaration of Helsinki and its later amendments.

### Randomisation

Eligible patients were randomly assigned (2:1) to either the experimental or control group. Randomisation was performed centrally using computer-generated randomisation lists.

### Chemoradiotherapy

Patients in both groups received concurrent chemoradiotherapy. All of the patients were treated with intensity-modulated radiotherapy (IMRT) using a linear accelerator with 6-MV X-rays *via* external beam radiation. The total dose prescribed to 95% volume PTV-GTV was 60–66Gy/30–33 times and PTV-CTV 46–50Gy/23–25 times. Chemotherapy consisted of docetaxel and cisplatin was administered every 21–28 days. The average chemotherapy cycles of the experimental group were 2.5 ± 1.2, while that of the control group were 2.3 ± 1.0, with no significant difference (p=0.125).

### Nutritional Intervention

The patients in the experimental group were administered a whole-course enteral nutrition management. The basic process is as follows: (1) nutritional risk screening with NRS-2002, nutritional assessment with PG-SGA; (2) enteral nutrition with oral nutritional supplement (ONS, Nutrison produced by Nutricia) or tube feeding based on the results of nutrition assessment, dietary investigation, degree of dysphagia; (3) timely evaluation of the treatment effect and adjustment of the nutritional program according to the dynamic changes of the nutritional status and adverse effect of patients; (4) quality control of the whole-course nutrition. The enteral nutrition was conducted by a nutrition support team (NST), which included clinicians, nutritionists, pharmacologists and nutrition nurses. The intake goals for energy and protein were set as 30–35 kcal/kg/d and 1.5–2.0g/kg/d, respectively. Doctors and nurses recorded and checked the patients’ energy and protein intake every day and ensured nutrition quality control, such that each patient received sufficient nutrients.

Whereas, the control group was treated with unsystematic nutrition based on the general eating conditions, hematologic test and treatment toxicities but not the nutritional assessment and dietary investigation, without considering the intake goals and nutrition quality control.

### Endpoints

The primary endpoint of the study was the change in body weight which was measured every week. Body weight change after the treatment = body weight evaluated within 1 week after treatment - body weight before the start of treatment. The secondary endpoints included: a) changes in haemoglobin and serum albumin levels, which were defined as haemoglobin and serum albumin levels evaluated within 1 week after treatment - haemoglobin and serum albumin levels before the start of treatment, monitored every week and at least every two weeks, respectively; b) side effects of radiotherapy and chemotherapy, which were evaluated according to the Radiation Therapy Oncology Group criteria and the National Cancer Institute Common Terminology Criteria for Adverse Events (version 3.0); c) infection rate, which was defined as patients with the use of antimicrobials; d) treatment completion rate; e) survival, including progression-free survival (PFS) and overall survival (OS).

### Follow-Up

All patients were followed up every 3 months within the first 3 years after treatment completion by outpatient clinic, telephone, WeChat, etc. After the third year, follow-up was performed every 6 months until 5 years after treatment completion. Contrast computed tomography of the chest, ultrasonography of the neck and abdomen, contrast esophagography and whole-body bone ECT scan were scheduled during follow-up. Additional diagnostic investigations, such as MRI, PET-CT and fine-needle aspiration, were carried out if recurrence was suspected by these routine examinations or if complaints, such as hoarseness, renewed dysphagia, unexplained weight loss or pain, arose before the next scheduled visit.

### Statistical Analyses

All statistical analyses were performed using the Statistical Package for the Social Sciences for Windows (software version 19.0; SPSS Inc., Chicago, IL, USA). Based on our preliminary experiment results, assuming a mean decrease in body weight of 0.70kg in the experimental group and 2.25 kg in the control group after treatment, a sample size of 177 patients was required (with a two-sided 5% significance level and a power of 80%). Accounting for an assumed drop-out rate of 20%, a target recruitment of 213 patients was established. Categorical variables were described by percentages and compared using the Chi-square test. Continuous variables were described by mean ± standard deviation (SD) and compared using two-sample Student’s *t*-test or analysis of variance (ANOVA), when appropriate. The PFS and OS curves were derived using the Kaplan-Meier method and compared using log-rank test. Univariable and multivariable Cox proportional hazards models was used to establish the effect of enteral nutrition in subgroups. A two-sided p-value < 0.05 was considered statistically significant.

## Results

### Baseline Characteristics of Patients

From March 2014 to June 2017, based on the established inclusion and exclusion criteria, a total of 222 esophageal squamous cell carcinomas patients from ten hospitals in China were randomised into the experimental (n=148) and control (n=74) groups. In the experimental group, 9 patients withdrew from the study, and 16 patients were lost during follow-up. A total of 10 patients in the control group withdrew from the study, and 7 patients were lost during follow-up. Therefore, in the final analysis, 123 patients in the experimental group and 57 in the control group were included. As summarised in **Table 1**, the baseline characteristics of the patients were similar in both the groups without statistical differences. A total of 27 (21.9%) patients in the experimental group had tube feeding, of which 20 patients were treated with nasogastric feeding tube and 7 patients used percutaneous endoscopic gastrostomy (PEG).

**Table 1:** Characteristics of patients in experimental and control groups at baseline.

Content	Experimental group (N=123)	Control group (N=57)	p-value
Age^a^			
<60 years	39 (31.7%)	21 (36.8%)	0.502^c^
≥60 years	84 (68.3%)	36 (63.2%)	
Gender^a^			
Male	94 (76.4%)	48 (84.2%)	0.326^c^
Female	29 (23.6%)	9 (15.8%)	
Clinical stage^a^			
II	21 (17.1%)	13 (22.8%)	0.414^c^
III	102 (82.9%)	44 (77.2%)	
Tumour length^a^			
<5cm	58 (47.2%)	23 (40.4%)	0.424^c^
≥5cm	65 (52.8%)	34 (59.6%)	
Median (cm)^b^	4.95±2.00	5.22±2.20	0.416^d^
KPS score^a^			
≤80	61 (49.6%)	32 (56.1%)	0.428^c^
>90	62 (50.4%)	25 (43.9%)	
PG-SGA^a^			
B	76 (61.8%)	34 (59.6%)	0.870^c^
C	47 (38.2%)	23 (40.4%)	
Tumor location^a^			
cervical	24 (19.5%)	11 (19.3%)	
upper thoracic	63 (51.2%)	27 (47.4%)	0.316^c^
middle thoracic	34 (27.6%)	15 (26.3%)	
lower thoracic	2 (1.7%)	4 (7.0%)	
Weight (kg)^b^	58.96±8.95	58.25±9.61	0.448^d^

KPS, Karnofsky Performance Status; PG-SGA, Patient-generated Subjective Global Assessment.

a Categorical variables were presented by number (%).

b Continuous variables were presented as mean ± standard deviation.

c Pearson chi-square test for categorical data was used.

d Two independent sample t-test for numeric variables data were used.

### Nutritional Status, Toxicities and Treatment Completion

The early results of nutritional status and toxicities of this study have previously been published in Chinese (13). Our re-analysis suggests that the average weight loss in the experimental group after the treatment was 0.73 ± 2.78 kg, which was significantly less than that in the control group (3.47 ± 3.78 kg). Participants in the experimental group showed less decline in serum albumin levels than in the control group (3.86 ± 4.95 vs. 6.03 ± 5.22 g/L, p=0.008) and haemoglobin (10.64 ± 13.61 g/L vs. 17.67 ± 15.42 g/L, p=0.002). Incidence of grade 3/4 leukopenia (45.6% vs. 27.6%, p=0.027) and infection rate (28.1% vs. 13.0%, p=0.020) was significantly frequent in the control than in the experimental group. Patients in the experimental group experienced higher chemoradiotherapy completion rates than those in control group (96.7% vs. 87.7%, p=0.038). Further, there were no significant inter-group differences in the lymphocyte count, ≥ G2 radiation pneumonitis and radiation esophagitis (p > 0.05) (**Table 2**).

**Table 2:** Comparison of nutritional status, toxicities and treatment completion between the experimental and control groups.

Content	Experimental group	Control group	p-value
Weight change after the treatment (kg)^b^	-0.73±2.78	-3.47±3.78	0.000^d^
Changes in haemoglobin after the treatment (g/L)^b^	-10.64±13.61	-17.67±15.42	0.002^d^
Changes in serum albumin after the treatment (g/L)^b^	-3.86±4.95	-6.03±5.22	0.008^d^
Changes in the lymphocyte count after the treatment (10^9^/L)^b^	-0.84±0.66	-0.94±0.80	0.361^d^
≥G3 leukopenia^a^	27.6%	45.6%	0.027^c^
≥G2 radiation pneumonitis^a^	27.6%	29.8%	0.859^c^
≥G2 radiation esophagitis^a^	34.1%	43.9%	0.247^c^
Incidence of infection^a^	13.0%	28.1%	0.020^c^
Treatment completion rate^a^	96.7%	87.7%	0.038^c^

a Categorical variables were presented as numbers (%).

b Continuous variables were presented as mean ± standard deviation.

c Pearson chi-square test for categorical data was used.

d Two independent sample t-test for numeric variables data were used.

### Overall Survival of the Patients

**Figure 1:**
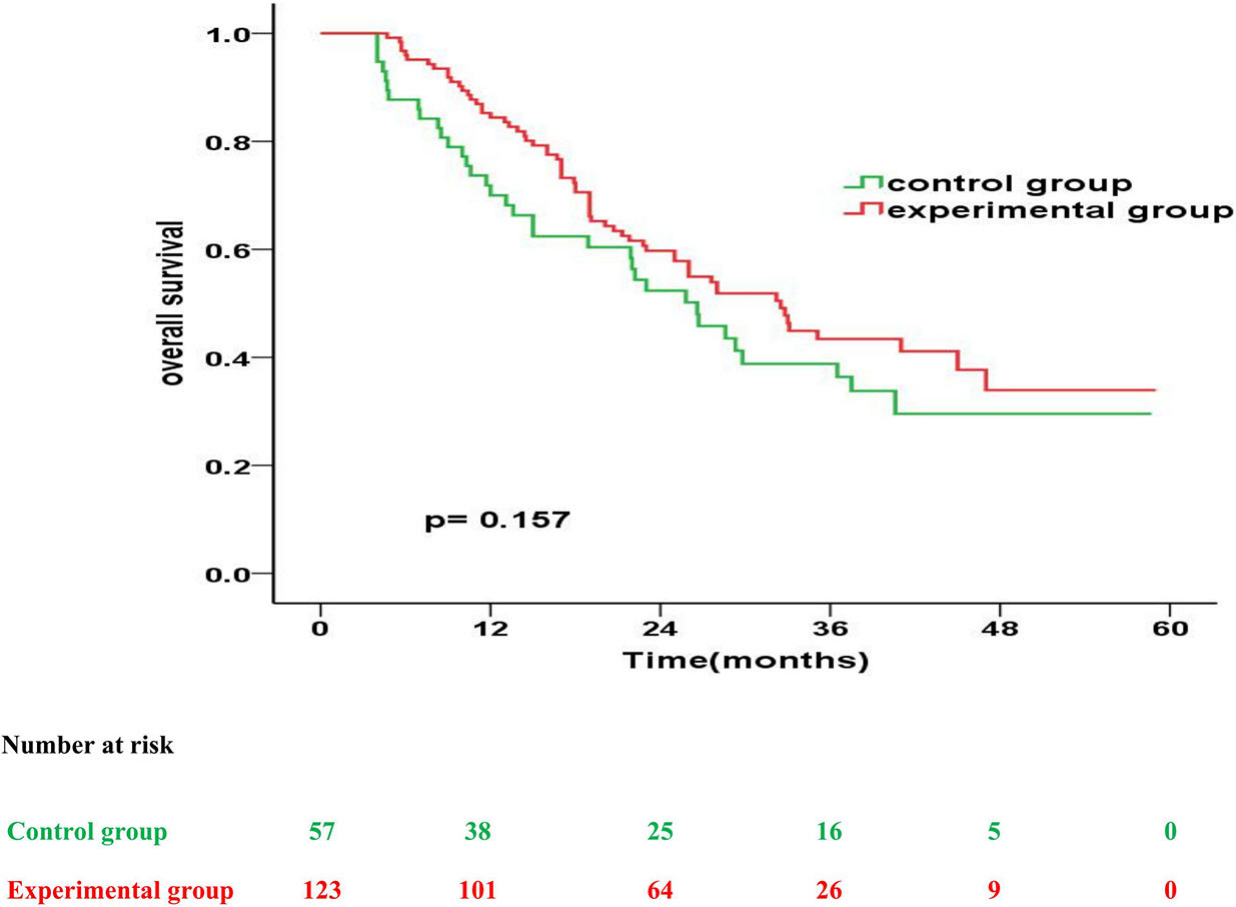
Kaplan-Meier overall survival curves for patients in the experimental group vs. control group.

The experimental and control group had a similar median OS (32.5 months vs. 26.6 months; p = 0.157). However, the OS rates in the experimental and control group were 83.6% and 70.0% at 1 year (p = 0.025), 58.9% and 52.3% at 2 years (p = 0.220), and 42.5% and 38.8% at 3 years (p = 0.323), respectively, after treatment (**Figure 1**).

**Figure 2:**
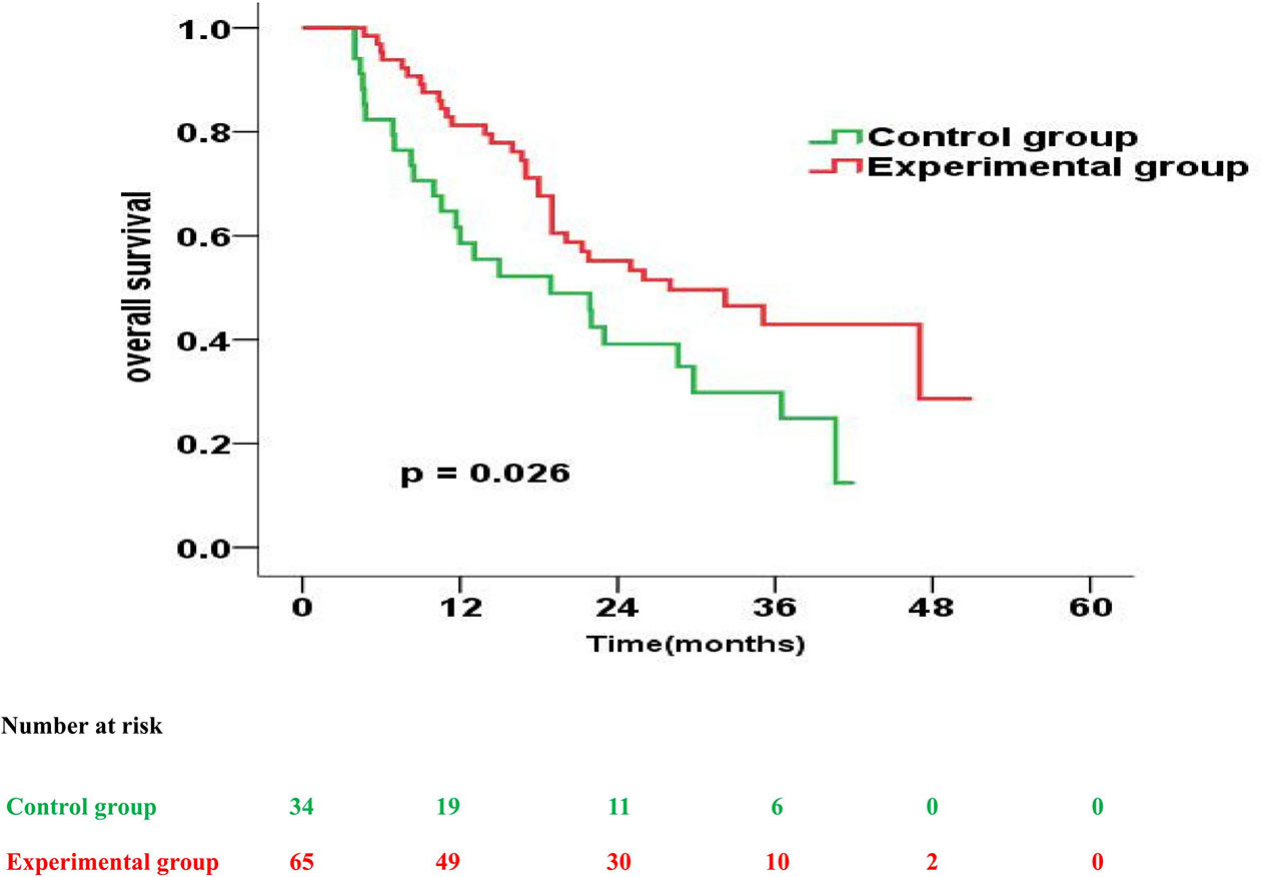
Overall survival curves for patients with tumour length ≥ 5cm in the experimental group vs. control group.

**Figure 3:**
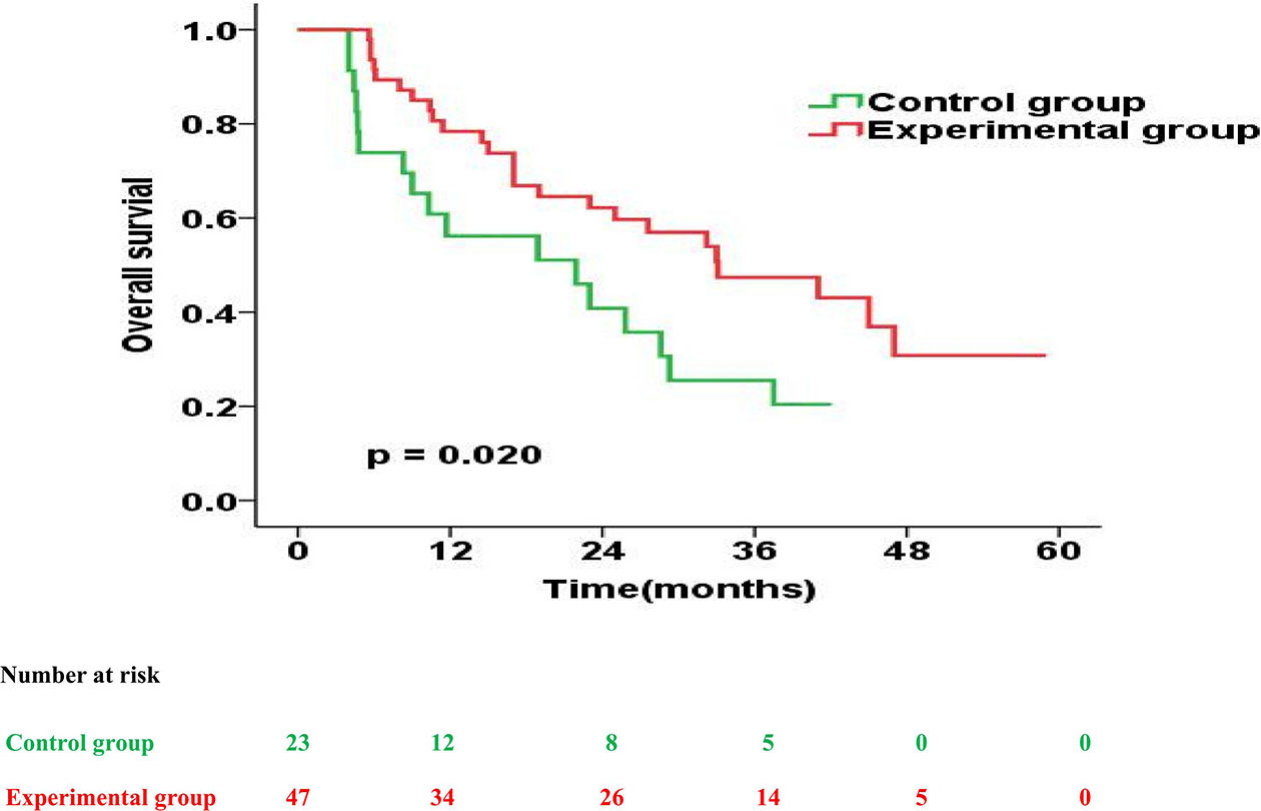
Overall survival curves for patients with PG-SGA qualitative evaluation = C in the experimental group vs. control group.

The prognostic impact of enteral nutrition on OS in subgroups of EC patients with different characteristics was analysed with the Cox proportional hazards regression model (**Table 3**). Patients with tumour length ≥ 5cm or PG-SGA=C had increased probability of better OS from enteral nutrition. Moreover, in patients with tumour length ≥ 5cm, the median OS was 28.0 months and 18.9 months in the experimental and control groups, respectively (p = 0.026) (**Figure 2**). Whereas, in patients with PG-SGA qualitative evaluation=C, the median OS was 33.1 months and 21.9 months in the experimental and control groups, respectively (p = 0.020) (**Figure 3**).

**Table 3:** Prognostic impact of enteral nutrition in subgroups of EC patients with different characteristics.

Subgroup	Univariate analysis		Multivariate analysis	
	HR (95% CI)	p-value	HR (95% CI)	p-value
Tumour length				
<5cm, experimental group vs. control group	1.169 (0.587 to 2.329)	0.656	1.022 (0.499 to 2.092)	0.952
≥5cm, experimental group vs. control group	0.553 (0.325 to 0.939)	0.028	0.544 (0.318 to 0.933)	0.027
PG-SGA qualitative evaluation				
B, experimental group vs. control group	0.955 (0.545 to 1.673)	0.871	0.842 (0.469 to 1.511)	0.564
C, experimental group vs. control group	0.481 (0.256 to 0.904)	0.023	0.458 (0.236 to 0.889)	0.021
Age				
<60 years, experimental group vs. control group	0.599 (0.295 to 1.217)	0.156	0.563 (0.268 to 1.180)	0.128
≥60 years, experimental group vs. control group	0.837 (0.503 to 1.395)	0.496	0.809 (0.483 to 1.355)	0.420
KPS score				
≤80 , experimental group vs. control group	0.633 (0.367 to 1.091)	0.100	0.586 (0.330 to 1.039)	0.067
>90, experimental group vs. control group	0.881 (0.461 to 1.682)	0.701	0.887 (0.462 to 1.702)	0.718
Clinical stage				
II, experimental group vs. control group	0.564 (0.217 to 1.466)	0.240	0.459 (0.165 to 1.278)	0.136
III, experimental group vs. control group	0.790 (0.498 to 1.254)	0.318	0.812 (0.509 to 1.295)	0.381
Gender				
Male	0.840 (0.529 to 1.333)	0.458	0.808 (0.507 to 1.287)	0.369
Female	0.556 (0.221 to 1.398)	0.212	0.444 (0.164 to 1.199)	0.109

KPS, Karnofsky performance status; PG-SGA, Patient-generated Subjective Global Assessment; CI, confidence interval; HR, hazard ratio.

### Progression-Free Survival of the Patients

**Figure 4:**
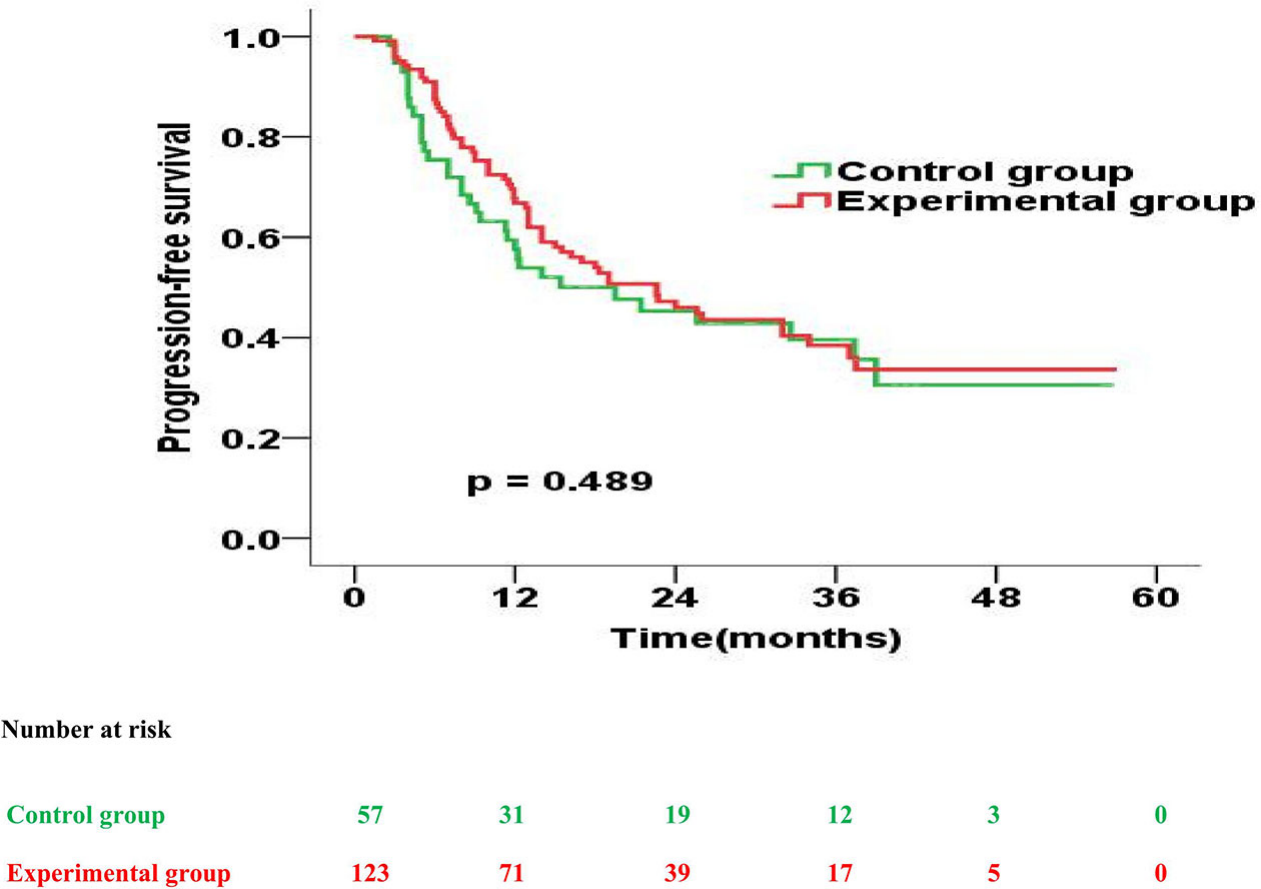
Progression-free survival curves for patients in the experimental group vs. control group.

Further, the experimental and control group had a similar median PFS (22.6 months vs. 19.5 months; p = 0.489). However, the 1-year, 2-year, 3-year PFS rates were 67.8% versus 57.6% (p = 0.135), 47.5% versus 46.0% (p = 0.573) and 41.5% versus 38.4% (p = 0.648) for patients treated in the experimental and control group, respectively (**Figure 4**).

**Figure 5:**
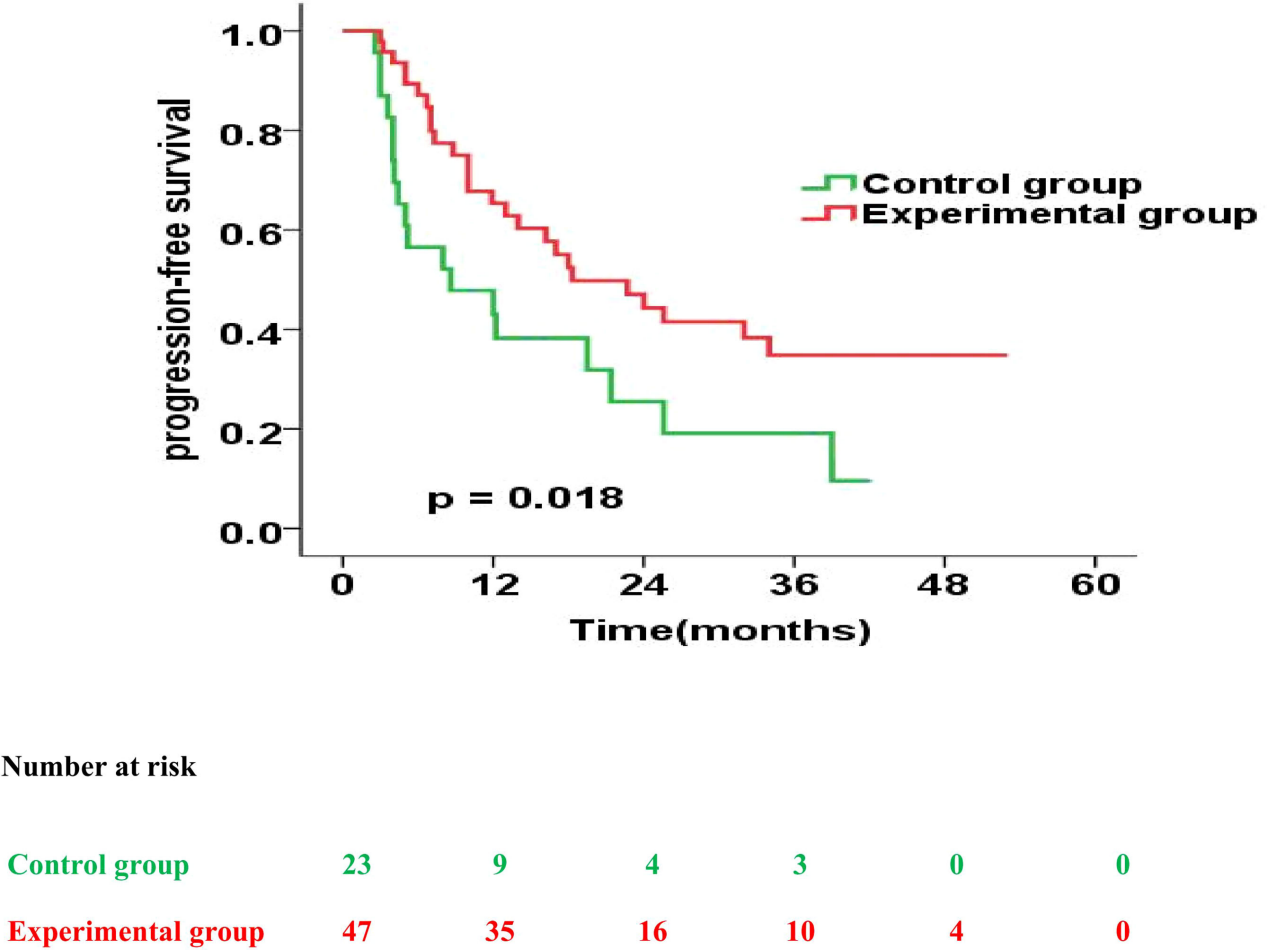
Progression-free survival curves for patients with PG-SGA qualitative evaluation = C in the experimental group vs. control group.

Next, we also analysed the prognostic impact of enteral nutrition on PFS in subgroups of ESCC patients with different characteristics using the Cox proportional hazards regression model (**Table 4**). Furthermore, in patients with PG-SGA qualitative evaluation=C in the experimental versus those in the control group, the median PFS was 18.3 months versus 8.6 months (p = 0.018) (**Figure 5**).

**Table 4:** Progression-free survival of enteral nutrition in subgroups of oesophageal squamous cell carcinoma patients with different characteristics.

Subgroup	Univariate analysis		Multivariate analysis	
	HR (95% CI)	p-value	HR (95% CI)	p-value
Tumour length				
<5cm, experimental group vs. control group	1.530 (0.747 to 3.134)	0.245	1.630 (0.763 to 3.480)	0.207
≥5cm, experimental group vs. control group	0.604 (0.360 to 1.014)	0.091	0.637 (0.378 to 1.075)	0.091
PG-SGA qualitative evaluation				
B, experimental group vs. control group	0.765 (0.425 to 1.375)	0.370	0.730 (0.398 to 1.336)	0.307
C, experimental group vs. control group	0.493 (0.269 to 0.902)	0.022	0.527 (0.285 to 0.972)	0.040
Age				
<60 years, experimental group vs. control group	0.738 (0.386 to 1.408)	0.356	0.750 (0.384 to 1.466)	0.401
≥60 years, experimental group vs. control group	0.963 (0.559 to 1.661)	0.892	0.991 (0.567 to 1.733)	0.975
KPS score				
≤80 , experimental group vs. control group	0.785 (0.457 to 1.347)	0.380	0.812 (0.464 to 1.423)	0.468
>90, experimental group vs. control group	1.097 (0.561 to 2.145)	0.786	1.079 (0.549 to 2.121)	0.824
Clinical stage				
II, experimental group vs. control group	1.223 (0.418 to 3.583)	0.713	1.787 (0.509 to 6.267)	0.365
III, experimental group vs. control group	0.780 (0.497 to 1.223)	0.279	0.870 (0.552 to 1.373)	0.550
Gender				
Male	0.930 (0.599 to 1.444)	0.747	0.912 (0.585 to 1.423)	0.685
Female	0.892 (0.237 to 3.361)	0.866	0.748 (0.177 to 3.164)	0.693

KPS, Karnofsky performance status; PG-SGA, Patient-generated Subjective Global Assessment; CI, confidence interval; HR, hazard ratio.

## Discussion

The prevalence of malnutrition is high among patients with oesophageal cancer, which negatively affects the therapy outcomes (4–6). The causes of malnutrition are complex, including psychological and mechanical reasons. Further, insufficient energy and protein intake caused by dysphagia, food avoidance, and diet change are the main mechanical reasons for malnutrition in the patients with oesophageal carcinoma (14). Therefore, in the present study, we intended to provide enteral nutrition to the patients with oesophageal cancer undergoing concurrent chemoradiothrapy (CCRT) to ensure sufficient energy and protein intake, thereby improving the nutritional status of the patients.

The change in body weight was considered as the primary endpoint since weight loss is a sensitive indicator of malnutrition, and a frequent cause of concern in patients and doctors. Moreover, a high prevalence of weight loss has been reported in oesophageal carcinoma patients at diagnosis and during the treatment (15). Additionally, Jiang et al. (6) reported 40.3% of oesophageal carcinoma patients to have ≥ 5% weight loss during radiotherapy. Furthermore, weight loss correlates with impairment of physical and psychological functions, low quality of life and poor prognosis. Therefore, maintenance of body weight in these patients is an important issue for clinicians globally.

Here, while patients in both control and experimental groups showed decrease in body weight during CCRT, those in the experimental group regained their weight after completion of treatment. Moreover, the average weight loss in the experimental group during and after CCRT was significantly less than that in the control group. The body weight alteration may be the result of an imbalance between energy intake and energy expenditure caused by reduction in food intake due to tumor obstruction, radiotherapy or chemotherapy-induced toxicities and cachexia-related high catabolism. Further, patients in the experimental group were administered a whole-course and systematic enteral nutrition, which effectively ensured their daily energy and protein needs. Thus, this strategy may be the possible explanation for better maintenance and quick recovery in body weight observed in patients in the experimental group.

Furthermore, our analysis also indicated that enteral nutrition could significantly reduce the declination in levels of serum albumin and haemoglobin, reduce the rates of grade 3/4 leukopenia and infection, and increase the chemoradiotherapy completion rates. Moreover, similar results have been reported in other studies. For instance, Odelli C. et al. (16) showed that nutrition intervention conferred a significantly positive effect on the nutritional status and tolerance of definitive chemoradiation treatment in patients with oesophageal carcinoma. Additionally, Cong et al. (11) administered nutrition treatment with the help of an interdisciplinary nutrition support team in oesophageal carcinoma patients receiving CCRT. Their analysis suggested that nutritional therapy could help in maintaining the nutritional status, improving the compliance of CCRT, and reducing the duration of hospital stay and in-patient costs. Further, by performing a whole-course nutritional management of patients with oesophageal carcinoma undergoing CCRT, Qiu et al. (12) observed an improvement in the levels of albumin and total protein, and quality of life, while reduction in the complications of radiation oesophagitis. Taken together, our study enhances the current understanding of the effect of enteral nutrition on the nutritional status of patients with oesophageal carcinoma treated with CCRT.

Further, since requirement of nutrition to improve or maintain patient’s body weight and nutritional status is unquestionable, studies are being conducted to understand whether it can improve the survival. However, it remains a controversial topic due to the limitations of related research and data analysis. Moreover, most of the clinical studies on nutritional therapy of cancer patients consider improvement in body weight and other nutritional indicators as observational end-points and only few studies have conducted long-term follow-up of patient survival. Klek S et al. (17) conducted a randomised clinical study to determine whether the post-operative use of enteral nutrition could influence survival in the patients diagnosed with stomach cancer. Their analysis suggested that the enteral nutrition group may have a low risk of mortality, especially during the first year after intervention, although, the long-term OS rates were found to be similar in both the groups (p = 0.663). Next, in a double-blind, randomised, controlled trial conducted by Buijs N et al. (18), the patients with head and neck cancer receiving enteral nutrition showed a significantly better OS (p = 0.019), disease-specific survival (p = 0.022) and locoregional recurrence-free survival (p = 0.027). However, no significant differences in the occurrence of distant metastases or second primary tumour were observed between the groups.

Further, to evaluate whether the use of enteral nutrition during hospitalization can influence the survival, we conducted a long-term follow-up after discharge of the patients, given the beneficial effect of enteral nutrition on the survival of oesophageal carcinoma patients. While we did not observe differences in survival benefit between the experimental and control groups, patients in the experimental group showed higher OS and PFS without statistical significance. Furthermore, our analysis suggested a significant survival benefit in the experimental than the control group at 1-year, but not at 2- and 3-years post treatment.

While it is expected that nutritional treatment should improve nutritional status and thus the OS of the patients, question would arise as to why enteral nutrition in our study conferred significant improvement only in the nutritional status and 1-year survival, but no survival benefit 2-years post treatment. The possible explanation for these findings may be a lack of home enteral nutrition, since malnutrition occurs not only during hospitalization, but also at home post treatment. Moreover, uncontrolled disease, oesophageal stricture and delayed side-effects of chemotherapy and radiotherapy present hurdles in the maintenance of proper nutrition in such patients. Baker ML et al. (19) observed that in patients after oesophago-gastric resection without home enteral nutrition and 3–6 months after hospital discharge, the oral intakes for energy and protein were adequate in only 55% and 77% patients, respectively, whereas 26% of the patients required rescue feeding. Moreover, as compared to baseline values, weight loss exceeding 5% (average 10.4%) was observed in 82–83% of the patients, 6-weeks post-surgery. Further, though the patients in our study received enteral nutrition during hospitalisation and their nutritional status improved during CCRT, however, they did not receive systematic and continuous nutrition monitoring, education and treatment at home. Additionally, correct nutrition concepts, good nutrition habits and standardised nutritional treatment methods appear to have gradually been ignored, forgotten or abandoned by the patients and family members post discharge from hospitalisation. Thus, the nutritional status may return to the same level in both the groups of patients. Therefore, the benefits of in-hospital enteral nutrition can only be maintained for a short duration, and the effects diminish with time as the initial decrease in risk of death in the experimental group was less and statistically insignificant.

Further, in case of oesophageal carcinoma, the importance of home nutrition is gradually gaining attention, especially in patients undergoing surgery. Several studies have shown that reasonable home nutrition therapy can improve the nutritional status, quality of life and effects of anti-tumour treatment in the patients (19–22). Moreover, our study indirectly establishes the importance of home nutrition in patients with oesophageal carcinoma undergoing CCRT with an unexpected finding.

Additionally, we observed an interesting finding in the subgroup analysis. Although our analysis did not validate the effect of enteral nutrition on the long-term survival in all patients, the OS and PFS benefit was observed among patients with PG-SGA=C. Thus, in overall, the beneficial effect of enteral nutrition was more evident in patients with worse nutrition status, and significantly less or even doubtful in other patients. Furthermore, consistent with our study, Qiu M et al. (23) observed that in stage IV gastric cancer patients who received chemotherapy and had NRS ≥ 3, the nutrition support could help in improving the prognosis. Thus, these results suggest that the benefits of nutritional therapy may vary among different populations. Therefore, we recommend that patients should be individually stratified to determine the requirement of nutritional treatment. The PG-SGA is an adaptation of the validated nutrition assessment tool—SGA, and has been specifically developed for utility in cancer patients. It has been commonly used to assess the patient’s nutritional status in clinical studies and has a significant correlation with the performance status and prognosis of patients with oesophageal carcinoma (24, 25). Moreover, the PG-SGA may be a useful reference index to determine nutritional treatment indications, although it is not the sole index. Additionally, we anticipate that this study may have important guiding significance for future research and clinical work.

## Conclusion

In conclusion, our study showed that whole-course enteral nutrition management can be beneficial for maintaining body weight and nutritional status of patients with oesophageal carcinoma receiving CCRT, and improving their treatment tolerance and short-term prognosis (especially the patients with PG-SGA=C). Additional follow-up is required to confirm the beneficial effect of EN support in long-term survival.

## Data Availability Statement

The original contributions presented in the study are included in the article/supplementary material. Further inquiries can be directed to the corresponding author.

## Ethics Statement

The studies involving human participants were reviewed and approved by Ethics Committee of Sichuan Cancer Hospital. The patients/participants provided their written informed consent to participate in this study. Written informed consent was obtained from the individual(s) for the publication of any potentially identifiable images or data included in this article.

## Author Contributions

TL, HS, and JHL contributed to the conception and design of the current work. TL and JHL contributed to data analyses and data interpretation. AS, JL, RZ, SZ, JHW, LX, DY, CX, LS, HZ, GZ, JW, WP, FL, and JYL substantially contributed to research conduction and data collection. All the authors have contributed to interpretation of data, and critically revised and approved the final version of this manuscript.

## Funding

This study was funded by the Wu Jieping medical foundation (320.6750.17237).

## Conflict of Interest

The authors declare that the research was conducted in the absence of any commercial or financial relationships that could be construed as a potential conflict of interest.

## Publisher’s Note

All claims expressed in this article are solely those of the authors and do not necessarily represent those of their affiliated organizations, or those of the publisher, the editors and the reviewers. Any product that may be evaluated in this article, or claim that may be made by its manufacturer, is not guaranteed or endorsed by the publisher.

## References

[1] Bray F Ferlay J Soerjomataram I Siegel RL Torre LA Jemal A. Global Cancer Statistics 2018: GLOBOCAN Estimates of Incidence and Mortality Worldwide for 36 Cancers in 185 Countries. CA Cancer J Clin (2018) 68(6):394–424. doi: 10.3322/caac.21492 30207593

[2] Zheng RS Sun KX Zhang SW Zeng HM Zou XN Chen R. Report of Cancer Epidemiology in China, 2015. Chin J Oncol (2019) 41(1):19–28. doi: 10.12968/nuwa.2015.19.28 30678413

[3] Bozzetti F Mariani L Lo Vullo S Amerio ML Biffi R Caccialanza G. The Nutritional Risk in Oncology: A Study of 1,453 Cancer Outpatients. Support Care Cancer (2012) 20(8):1919–28. doi: 10.1007/s00520-012-1387-x PMC3390688 22314972

[4] Lloyd S Chang BW. Current Strategies in Chemoradiation for Esophageal Cancer. J Gastrointest Oncol (2014) 5(3):156–65. doi: 10.3978/j.issn.2078-6891.2014.033 PMC4074950 24982764

[5] Song C Cao J Zhang F Wang C Guo Z Lin Y. Nutritional Risk Assessment by Scored Patient-Generated Subjective Global Assessment Associated With Demographic Characteristics in 23,904 Common Malignant Tumors Patients. Nutr Cancer (2019) 71(1):50–60. doi: 10.1080/01635581.2019.1566478 30741002

[6] Jiang N Zhao JZ Chen XC Li LY Zhang LJ Zhao Y. Clinical Determinants of Weight Loss in Patients With Esophageal Carcinoma During Radiotherapy: A Prospective Longitudinal View. Asian Pac J Cancer Prev (2014) 15(5):1943–8. doi: 10.7314/apjcp.2014.15.5.1943 24716916

[7] Mak M Bell K Ng W Lee M. Nutritional Status, Management and Clinical Outcomes in Patients With Esophageal and Gastro-Oesophageal Cancers: A Descriptive Study. Nutr Diet (2017) 74(3):229–35. doi: 10.1111/1747-0080.12306 28731604

[8] Clavier JB Antoni D Atlani D Ben Abdelghani M Schumacher C Dufour P. Baseline Nutritional Status Is Prognostic Factor After Definitive Radiochemotherapy for Esophageal Cancer. Dis Esophagus (2014) 27(6):560–7. doi: 10.1111/j.1442-2050.2012.01441.x 23106980

[9] Miyata H Yano M Yasuda T Hamano R Yamasaki M Hou E. Randomized Study of Clinical Effect of Enteral Nutrition Support During Neoadjuvant Chemotherapy on Chemotherapy-Related Toxicity in Patients With Esophageal Cancer. Clin Nutr (2012) 31(3):330–6. doi: 10.1016/j.clnu.2011.11.002 22169459

[10] Arends J Bodoky G Bozzetti F Fearon K Muscaritoli M Selga G. ESPEN Guidelines on Enteral Nutrition: Non-Surgical Oncology. Clin Nutr (2006) 25(2):245–59. doi: 10.1016/j.clnu.2006.01.020 16697500

[11] Cong MH Li SL Cheng GW Liu JY Song CX Deng YB. An Interdisciplinary Nutrition Support Team Improves Clinical and Hospitalized Outcomes of Esophageal Cancer Patients With Concurrent Chemoradiotherapy. Chin Med J (2015) 128(22):3003–7. doi: 10.4103/0366-6999.168963 PMC4795249 26608978

[12] Qiu Y You J Wang K Cao Y Hu Y Zhang H. Effect of Whole-Course Nutrition Management on Patients With Esophageal Cancer Undergoing Concurrent Chemoradiotherapy: A Randomized Control Trial. Nutrition (2020) 69:110558. doi: 10.1016/j.nut.2019.110558 31526964

[13] Lyu JH Li T Zhu GY Li J Zhao R Zhu SC. Influence of Enteral Nutrition on Nutritional Status, Treatment Toxicities, and Short⁃Term Outcomes in Esophageal Carcinoma Patients Treated With Concurrent Chemoradiotherapy: A Prospective, Multicenter, Randomized Controlled Study (NCT 02399306). Chin J Radiat Oncol (2018) 27(1):44–8. doi: 10.3760/cma.j.issn.1004-422

[14] Miller KR Bozeman MC. Nutrition Therapy Issues in Esophageal Cancer. Curr Gastroenterol Rep (2012) 14(4):356–66. doi: 10.1007/s11894-012-0272-6 22730015

[15] Hill A Kiss N Hodgson B Crowe TC Walsh AD. Associations Between Nutritional Status, Weight Loss, Radiotherapy Treatment Toxicity and Treatment Outcomes in Gastrointestinal Cancer Patients. Clin Nutr (2011) 30(1):92–8. doi: 10.1016/j.clnu.2010.07.015 20719409

[16] Odelli C Burgess D Bateman L Hughes A Ackland S Gillies J. Nutrition Support Improves Patient Outcomes, Treatment Tolerance and Admission Characteristics in Oesophageal Cancer. Clin Oncol (2005) 17(8):639–45. doi: 10.1016/j.clon.2005.03.015 16372491

[17] Klek S Scislo L Walewska E Choruz R Galas A. Enriched Enteral Nutrition may Improve Short-Term Survival in Stage IV Gastric Cancer Patients: A Randomized, Controlled Trial. Nutrition (2017) 36:46–53. doi: 10.1016/j.nut.2016.03.016 28336107

[18] Buijs N van Bokhorst-de van der Schueren MA Langius JA Leemans CR Kuik DJ Vermeulen MA. Perioperative Arginine-Supplemented Nutrition in Malnourished Patients With Head and Neck Cancer Improves Long-Term Survival. Am J Clin Nutr (2010) 92(5):1151–6. doi: 10.3945/ajcn.2010.29532 20881073

[19] Baker ML Halliday V Robinson P Smith K Bowrey DJ. Nutrient Intake and Contribution of Home Enteral Nutrition to Meeting Nutritional Requirements After Oesophagectomy and Total Gastrectomy. Eur J Clin Nutr (2017) 71(9):1121–8. doi: 10.1038/ejcn.2017.88 PMC5590712 28656968

[20] Donohoe CL Healy LA Fanning M Doyle SL Hugh AM Moore J. Impact of Supplemental Home Enteral Feeding Postesophagectomy on Nutrition, Body Composition, Quality of Life, and Patient Satisfaction. Dis Esophagus (2017) 30(9):1–9. doi: 10.1093/dote/dox063 28859364

[21] Li X Zhou J Chu C You Q Zhong R Rao Z. Home Enteral Nutrition may Prevent Myelosuppression of Patients With Nasopharyngeal Carcinoma Treated by Concurrent Chemoradiotherapy. Head Neck (2019) 41(10):3525–34. doi: 10.1002/hed.25861 31301097

[22] Ruggeri E Giannantonio M Agostini F Ostan R Pironi L Pannuti R. Home Artificial Nutrition in Palliative Care Cancer Patients: Impact on Survival and Performance Status. Clin Nutr (2020) 39(11):3346–53. doi: 10.1016/j.clnu.2020.02.021 32143890

[23] Qiu M Zhou YX Jin Y Wang ZX Wei XL Han HY. Nutrition Support can Bring Survival Benefit to High Nutrition Risk Gastric Cancer Patients Who Received Chemotherapy. Support Care Cancer (2015) 23(7):1933–9. doi: 10.1007/s00520-014-2523-6 25492636

[24] Quyen TC Angkatavanich J Thuan TV Xuan VV Tuyen LD Tu DA. Nutrition Assessment and Its Relationship With Performance and Glasgow Prognostic Scores in Vietnamese Patients With Esophageal Cancer. Asia Pac J Clin Nutr (2017) 26(1):49–58. doi: 10.6133/apjcn.122015.02 28049261

[25] Lyu J Li T Xie C Li J Xing L Zhang X. Enteral Nutrition in Esophageal Cancer Patients Treated With Radiotherapy: A Chinese Expert Consensus 2018. Future Oncol (2019) 15(5):517–31. doi: 10.2217/fon-2018-0697 30457348

